# Behandlungsstrategien und Ergebnisse bei injektionsassoziierten inguinalen perivaskulären Abszessen bei intravenös Drogenabhängigen

**DOI:** 10.1007/s00104-020-01137-3

**Published:** 2020-02-14

**Authors:** D. Liebetrau, E. Feder, S. Zerwes, Y. Goßlau, A. Hyhlik-Dürr

**Affiliations:** 1 Klinik für Gefäßchirurgie und endovaskuläre Chirurgie, Universitätsklinikum Augsburg, Stenglinstraße 2, 86156 Augsburg, Deutschland; 2 Klinik Günzburg, Ludwig-Heilmeyer-Straße 1, 89312 Günzburg, Deutschland

**Keywords:** Leisteninfektion, Intravenöser Drogenabusus, Septische Arrosionsblutung, Leistenabszess, Gefäßkomplikation, Groin infection, Intravenous drug abuse, Septic arrosion bleeding, Groin abscess, Vascular complication

## Abstract

**Hintergrund:**

Langzeitdrogenabhängige präsentieren sich nach dem Verbrauch der oberflächlichen Venen regelmäßig mit tiefen inguinalen vaskulär assoziierten Abszessen infolge fortgesetzter Drogeninjektionen. Die Behandlung dieser Komplikationen stellt anhaltend eine große medizinische Herausforderung dar. Bisher werden keine einheitlichen Therapieregime in der Literatur beschrieben.

**Fragestellung:**

Welche Behandlungsstrategien und Ergebnisse gibt es bei injektionsassoziierten inguinalen perivaskulären Abszessen bei Drogenabhängigen?

**Material und Methoden:**

Die Daten aller im Zeitraum zwischen 01.01.2004 und 31.05.2019 am Universitätsklinikum Augsburg behandelten Drogenkonsumenten wurden retrospektiv aufgearbeitet und mit der vorliegenden Literatur verglichen.

**Ergebnisse:**

Es konnten 37 Fälle (männlich = 25, weiblich = 12) nach Anwendung der Einschlusskriterien in die Datenerhebung eingeschlossen werden. Das mediane Alter im untersuchten Patientenkollektiv lag bei 34,3 Jahren. Die 30-Tage-Mortalität lag bei 2,7 % (1/37). Die Amputationsrate betrug 2,8 %. Im untersuchten Kollektiv lag bei 13 Patienten eine arterielle Beteiligung vor. In 5 Fällen wurde primär arteriell ligiert. Bei weiteren 5 Fällen wurde primär eine Rekonstruktion mittels autologen Interponats durchgeführt. In weiteren 3 Fällen erfolgte die Anlage eines Obturatorbypasses (1/3) sowie die Durchführung einer Patchplastik (2/3). Die Offenheitsrate nach arterieller Rekonstruktion lag bei 87,5 % bei einem mittleren Follow-up von 421 Tagen. Die Gesamtkomplikationsrate lag bei 51,4 %.

**Diskussion:**

Bei vaskulärer Beteiligung ist ein situationsgerechtes Vorgehen sinnvoll. Neben der Beseitigung komplizierter, septisch-venöser Thrombosen erscheint die Korrektur arterieller Blutungen mittels autologer Rekonstruktionsmaßnahmen aussichtsreich.

## Abstract (en)

**Background:**

Long-term drug users regularly present with deep inguinal vascular-associated abscesses due to continued drug injections utilizing superficial veins. The treatment of these complications continues to be a major medical challenge. So far no uniform treatment regimens have been described in the literature.

**Objective:**

What are the treatment strategies and outcomes of injection-associated inguinal perivascular abscesses in drug addicts?

**Material and methods:**

All drug users treated at the Augsburg University Hospital in the period between 1 January 2004 and 31 May 2019 were retrospectively reviewed and compared with the currently available literature.

**Results:**

In this study 37 cases (male = 25, female = 12) could be included in the data collection after implementation of the inclusion criteria. The median age in the investigated patient population was 34.3 years. The 30-day mortality was 2.7% (1/37). The amputation rate was 2.8%. In the investigated collective 13 patients had arterial involvement, in 5 cases a ligature of arteries was primarily used and in another 5 cases a reconstruction using an autologous conduit graft was primarily performed. In another 3 cases an obturator bypass (1/3) was placed and a patch plasty (2/3). The patency rate after arterial reconstruction was 87.5% with a mean follow-up of 421 days. The overall complication rate was 51.4%.

**Conclusion:**

For vascular involvement an approach appropriate for the situation is meaningful. In addition to the elimination of complicated septic venous thromboses, the correction of arterial hemorrhages using autologous reconstruction measures seems promising.

Insbesondere in städtischen Ballungsgebieten werden Krankenhäuser und behandelnde Ärzte mit Komplikationen des intravenösen Drogenmissbrauchs konfrontiert. Dazu gehören injektionsassoziierte vaskuläre Komplikationen, welche aufgrund ihrer häufig fulminanten Verläufe von übergeordneter Bedeutung sind. Ein einheitlich anerkanntes Therapieregime zur Behandlung vaskulärer Komplikationen existiert nicht. In diesem Beitrag soll anhand von 37 Fällen evaluiert werden, welches chirurgische Vorgehen bei injektionsassoziierten inguinalen perivaskulären Abszessen am aussichtsreichsten ist.

## Hintergrund

Laut dem „World Drug Report“ der Vereinten Nationen haben im Jahr 2017 271 Mio. Menschen Drogen konsumiert. Insgesamt stellt dies im Vergleich zu 2009 einen Anstieg von 5,5 % dar. Die am meisten konsumierte Droge ist Cannabis. Zusätzlich gewinnen harte Drogen wie Kokain und Heroin und der damit verbundene intravenöse Drogenmissbrauch zunehmend an Bedeutung [1]. Durch einen langjährigen intravenösen (i.v.) Drogenkonsum kommt es im Laufe der Zeit zu Vernarbungen im Bereich der oberflächlichen Venen. Als alternativer Zugang werden dann bevorzugt die großen, tiefliegenden Gefäße der unteren Extremität gewählt [18].

Dabei fördert die Verwendung verunreinigter, kontaminierter Injektionsnadeln bakterielle Infektionen. Ausgedehnte Abszedierungen können auftreten und gehen mit einem hohen Risiko schwerwiegender Gefäßkomplikationen einher. Die Behandlung dieser Komplikationen stellt anhaltend eine große medizinische Herausforderung dar.

## Zielsetzung der Arbeit

Nach Recherchen in der medizinischen Fachliteratur existiert keine einheitliche Vorgehensweise zur Behandlung perivaskulärer Inguinalabszesse nach intravasaler Drogeninjektion. Zielsetzung dieser Arbeit ist es, einen Behandlungspfad sowie mögliche diagnostische Maßnahmen und chirurgische Therapiestrategien bei inguinalen Abszessen durch intravenösen Drogenabusus darzustellen.

## Patientenkollektiv und Methoden

Im Zeitraum 01.01.2004 bis 01.05.2019 wurden alle am Universitätsklinikum Augsburg behandelten Drogenkonsumenten mit Abszedierung der Inguinalregion in einer prospektiven Datenbank (Microsoft-Excel®-Version 2010; Microsoft Corporation, Redmond, WA, USA) erfasst und retrospektiv ausgewertet. Eingeschlossen wurden alle Patienten, die sich mit einem dokumentierten aktiven oder anamnestisch erhobenen Drogenabusus und einer Leisteninfektion vorstellten. Es konnten 37 Fälle (25 männliche, 12 weibliche Patienten) nach Anwendung der Einschlusskriterien in die Datenerhebung eingeschlossen werden.

Für die retrospektive Datenerhebung wurden die Krankenakten bzw. Mikrofilme und Operationsberichte zur Analyse herangezogen. Bei allen Patienten wurde bei jedem Aufenthalt eine virologische Diagnostik mittels HIV-Antikörpersuchtest-Diagnostik und Hepatitisserologie veranlasst. Als Komplikation wurde das Auftreten einer Blutung, eines akuten Nierenversagens, einer Nervenläsion, einer Pneumonie oder Ischämie der unteren Extremität im Rahmen des stationären Aufenthaltes definiert. Aufgrund des unterschiedlichen Infektionsausmaßes wurde das Patientenkollektiv in 4 Gruppen eingeteilt: 14 Fälle, bei denen keine Gefäßbeteiligung vorlag, 10 Fälle mit rein venöser Beteiligung, 11 Fälle mit rein arterieller und 2 Fälle mit einer kombiniert arteriovenösen Beteiligung.

## Ergebnisse

### Patientencharakteristika

Das mediane Alter im untersuchten Patientenkollektiv lag bei 34,3 Jahren. Zum Zeitpunkt der Aufnahme zeigten sich im Durchschnitt eine erhöhte Leukozytenzahl von 15,3 ± Standardabweichung (SD) 6,8/nl und ein C‑reaktives Protein (CRP) von 19,6 ± (SD) 9,6 mg/dl. Weiterhin zeigte sich bei 20 Personen zum Zeitpunkt des Erstkontakts Fieber mit einer Durchschnittstemperatur von 38,5° ± (SD) 0,65. Die mittlere Aufenthaltsdauer der Patienten betrug 35,1 ± (SD) 21,6 Tage. Die 30-Tage-Mortalität lag bei 2,7 % (1/37). Ein Patient verstarb an einem septischen Multiorganversagen. Bei einem Patienten musste aufgrund des ausgeprägten Weichteilinfektes eine Major-Amputation durchgeführt werden. Dies entspricht einer Amputationsrate von 2,8 % (Tab. 1). Im untersuchten Kollektiv lag bei 13 Patienten eine arterielle Beteiligung vor (Tab. 2). In 5 Fällen wurde primär arteriell ligiert. Bei weiteren 5 Fällen wurde primär eine Rekonstruktion mittels autologen Interponats durchgeführt. In weiteren 3 Fällen erfolgte die Anlage eines Obturatorbypasses (1/3) sowie die Durchführung einer Patchplastik (2/3). Zwei der 5 primär ligierten Patienten entwickelten im stationären Verlauf eine Extremitätenischämie und wurden sekundär einer Revaskularisation mittels autologen Interponats unterzogen. Insgesamt konnte bei 5/8 Patienten (arterielle Rekonstruktionen) ein Follow-up erhoben werden. Die Offenheitsrate nach arterieller Rekonstruktion lag bei 87,5 % bei einem mittleren Follow-up von 421 Tagen. Die Gesamtkomplikationsrate lag bei 51,4 % (Tab. 3).

**Table 1:** 

Patientenkollektiv	Gesamt (*n*)	(%)
Einbezogene Patienten	37	100,0
Geschlecht (m/w)	25|12	67,6|32,4
Alter in Jahren (MW ± SD)	35,7 ± 7,2	–
Median des Alters	34,3	–
Leukozyten/nl (MW ± SD)	15,3 ± 6,8	–
CRP mg/dl (MW ± SD)	19,6 ± 9,6	–
Fieber	27	73,0
Tage ITS (MW ± SD)	3,2 ± 7,2	–
HIV	1	2,7
Hepatitis C	25	67,6
Polytoxikomanie	33	89,2
Alkoholabusus	7	18,9
Nikotinabusus	30	81,1
Dauer KH-Aufenthalt (Tage)	35,1 ± 21,6	–
Durchgeführte Major-Amputationen	1	2,8

*MW* Mittelwert, *SD* Standardabweichung, *CRP* C-reaktives Protein, *ITS* Intensivstation, *HIV* Humane-Immundefizienz-Virus, *KH* Krankenhaus

**Table 2:** 

	Primär^b^ (*n*)	Sekundär^c^ (*n*)
*Einbezogene Patienten*	13	5
*Ligatur der Arterie*	5	2
*Interponat*		
Autolog (Vene)	5	3
Alloplastisch	–	1
*Extraanatomischer Bypass*	1	–
*Patch*		
Autolog (Vene)	1	–
Bovin	1	–
*Ischämiesymptomatik nach arterieller Ligatur*	2	–
*ABI*^*a*^* nach arterieller Ligatur (MW* *±* *SD)*	*0,65* *±* *0,24*	
*Follow-up arterieller Rekonstruktionen (Tage)*	*421*	
*Offenheitsrate arterieller Rekonstruktionen*	*87,5* *%*	

^a^Erhobener Ankle-brachial-Index (*ABI*) bei Entlassung

^b^Primäre Versorgung bei arterieller Gefäßbeteiligung

^c^Sekundäre Versorgung nach arterieller Ligatur, Interponat oder Patchplastik

**Table 3:** 

Patientenkollektiv		Gesamt		Ohne Gefäßbeteiligung		Venöse Gefäßbeteiligung		Arterielle Gefäßbeteiligung		Arterielle + venöse Gefäßbeteiligung	
		(*n*)	(%)	(*n*)	(%)	(*n*)	(%)	(*n*)	(%)	(*n*)	(%)
*Einbezogene Patienten*		37	100	14	37,8	10	27,0 €	11	29,7	2	5,4
NPWT		29	–	11	78,6	8	80,0	8	72,7	2	100,0
Nadelfragmente		4	–	1	7,1	1	0,0	1	9,1	1	50,0
Revisionen		111	–	27	24,3	15	13,5	54	48,6	15	13,5
Biologische Sicherung		9	–	2	14,3	0	0,0	6	54,5	1	50,0
Spalthautdeckung		10	–	3	21,4	2	20,0	4	36,4	1	50,0
Primäre Hautnaht		1	–	0	0,0	1	10,0	0	0,0	0	0,0
Sekundärnaht		7	–	2	14,3	3	30,0	1	9,1	1	50,0
Sekundärheilung		19	–	9	64,3	4	40,0	6	54,5	0	0,0
*Komplikationen*		*19*	*51,4*	*3*	*21,4*	*4*	*40,0*	*10*	*90,9*	*2*	*100,0*
Blutung		7	–	1	7,1	2	20,0	4	36,4	0	0,0
ANV		3	–	1	7,1	0	0,0	2	18,2	0	0,0
Pneumonie		4	–	1	7,1	1	10,0	1	9,1	1	50,0
Nervenläsion		3	–	0	0,0	1	10,0	2	18,2	0	0,0
Ischämie^a^		2	–	0	0,0	0	0,0	1	9,1	1	50,0
*Polamidon (ml/Tag)*^b^		7,9	–	7,4	–	7	–	8,5	–	12	–
*Beinerhalt bei*		36	–	14	38,9	10	27,8	10	27,8	2	5,6

*ANV* akutes Nierenversagen, *NPWT* „negative pressure wound therapy“

^a^Untere Extremitätenischämie

^b^Durchschnittlicher Verbrauch pro Tag

### Diagnostischer Algorithmus

**Figure 1:**
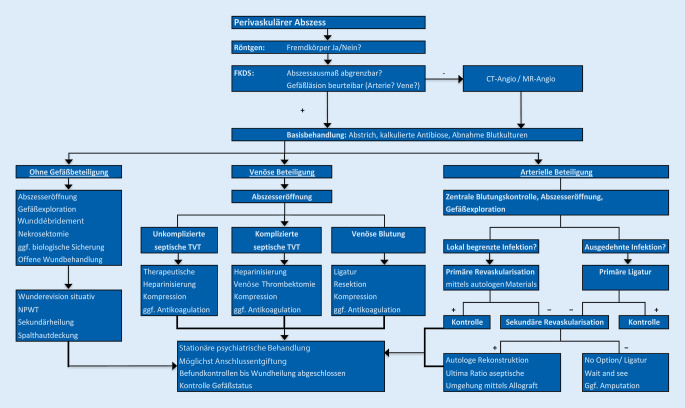

Alle injektionsassoziierten Lokalbefunde wurden zunächst einer farbkodierten duplexsonographischen Diagnostik zur Beurteilung der Abszessformation, Gefäßbeteiligung sowie Flussbeurteilung in den Femoralgefäßen unterzogen. In 13 Fällen erfolgte eine Röntgen(RT)-Übersichtsaufnahme zur Detektion eines Fremdkörpers. In 22 Fällen wurde bei nicht ausreichender Erfassung durch die Duplexsonographie aufgrund weitreichender teilweise multilokulärer inguinaler und retroperitonealer Abszedierungen sowie nicht sicherer Beurteilung der Gefäßsituation zusätzlich eine computertomographische Angiographie (CTA; SOMATOM Definition Flash, Siemens: 3 mm Schichtdicke) präoperativ durchgeführt. Die Kombination verschiedener Untersuchungen war möglich. Während des stationären Aufenthaltes erfolgte bei allen Patienten die konsiliarische Mitbeurteilung durch die Kollegen der Psychiatrie und bei Bedarf die Erstellung eines individuellen Substitutionsprogramms mittels Polamidon (Abb. 1).

## Diskussion

Die Altersverteilung lag in der zu erwartenden Spannbreite zwischen 22 und 50 Jahren. Es wurden mit 67,7 % deutlich häufiger Männer als Frauen mit dem komplexen Krankheitsbild eines injektionsassoziierten Leistenabszess behandelt. Dies korreliert mit den Angaben der Rauschgiftdatei des Bundeskriminalamts von 2017, indem die registrierten Drogentoten bei einem Durchschnittsalter von 39 Jahren zu 85 % männlich waren [3]. Ein Patient verstarb während des stationären Aufenthaltes und bei nur einem Patienten musste eine Major-Amputation durchgeführt werden. In unserem Patientenkollektiv konnte in 97,2 % (35/36) ein amputationsfreies Überleben erreicht werden. Insgesamt ergab sich trotz des jungen Patientenalters eine durchschnittliche stationäre Verweildauer von 35,1 ± 21,6 Tagen. Die hohe Gesamtkomplikationsrate von 51,4 % unterstreicht die hohe Morbidität der inguinalen Weichteilinfektionen.

### Präoperative Diagnostik

Auffällig ist, dass in den primär durchgeführten duplexsonographischen Befunden in keinem Fall der Verdacht auf das Vorliegen eines Spritzenfragmentes beschrieben wurde. Es konnte jedoch in insgesamt 4 Kasuistiken ein abgebrochenes Nadelfragment radiologisch nachgewiesen werden. Drei wurden dabei durch eine Beckenübersichtsaufnahme und eine primär in der CT-Angiographie diagnostiziert. Die Fremdkörper wurden intraoperativ unter Durchleuchtung geborgen. Die explizite Abklärung diesbezüglich ist für den Eigenschutz unabdingbar und besitzt höchste Priorität. Eine Untersuchung aus Großbritannien konnte zeigen das 20 % der i.v. Drogenabhängigen Erfahrungen mit abgebrochenen Nadeln gemacht haben [16]. Daher sollte zwingend ein radiologischer Ausschluss von Fremdkörpern zum Eigenschutz erfolgen (Abb. 1).

### Operative Ergebnisse

Bei der Behandlung komplizierter Inguinalabszesse mit Beteiligung der Femoralgefäße ist in der Literatur kein einheitliches Therapiekonzept beschrieben. Als allgemein gültige Strategie bei der Behandlung von Spritzenabszessen wird die ausgedehnte chirurgische Infektspaltung mit Abszesseröffnung und weitreichendem Weichteildébridement sowie anschließender konsequenter offener Wundbehandlung akzeptiert [9, 19, 22]. Die relativ kleine Fallzahl von 37 Inguinalabszessen ergibt sich daraus, dass keine oberflächlichen bzw. subkutanen Abszedierungen, sondern nur komplikationsträchtige Infektionen, die bis zu den Femoralgefäßen reichten, untersucht wurden. Verglichen mit anderen Veröffentlichungen [2, 9, 13, 14] ist das in dieser Arbeit vorgestellte Patientenkollektiv mit insgesamt 37 Fällen eines der größten deutschen untersuchten Kollektive.

Grundsätzlich zeigten sich im untersuchten Kollektiv 4 unterschiedliche Beteiligungsmuster der Femoralgefäße (Tab. 3). Dabei kommen unterschiedliche Behandlungsstrategien zum Tragen. Das therapeutische Vorgehen richtete sich bei Patienten mit rein venöser Beteiligung neben der chirurgische Infektspaltung nach der klinischen Beherrschbarkeit des lokalen Infektes (Tab. 4). Bei unkomplizierten Becken-Bein-Venenthrombose erfolgte die Therapie leitliniengerecht mittels therapeutischer Heparinisierung und Kompressionstherapie [2]. Die erfolgreiche Therapie der komplizierten/septischen iliakofemoralen Phlebothrombose kann häufig durch alleinige Heparinisierung und testgerechte Antibiotikatherapie erzielt werden [11]. In unserem untersuchten Kollektiv konnte in 6 Fällen ein konservatives Prozedere bei septischer Phlebothrombose erfolgreich durchgeführt werden. In weiteren 6 Fällen war unter konservativer Therapie keine Beherrschung des Infektgeschehens möglich. Es erfolgte daraufhin die chirurgische Therapie mittels Thrombektomie der V. femoralis communis (VFC, 1/6), Crossektomie der V. saphena magna mit Thrombektomie der VFC (1/6) oder Ligatur der VFC (4/6; Tab. 4). Die Thrombektomie der VFC wurde in der Literatur bei fortschreitender septischer oder zentraler Thrombosierung, multifokaler Abszedierung oder Embolisation bereits beschrieben [10]. Da die operative Behandlung der septischen Thrombose mittels offener venöser Thrombektomie äußerst komplikationsträchtig ist, sollte sie nur bei nicht beherrschbarer Sepsis erfolgen [7]. Weiterhin konnte eine hohe Komplikationsrate (90,9 %) in der Gruppe der Leistenabszesse mit arterieller Gefäßbeteiligung erhoben werden. Eine Möglichkeit, welche mit einem geringen Komplikationsrisiko hinsichtlich nachfolgender septischer Arrosionsblutung und postoperativer Letalität behaftet ist, stellt die primäre Ligatur der Leistenarterien dar. Dies gilt vor allem für isolierte Gefäßläsionen unterhalb der Femoralisgabel im Bereich der A. femoralis superficialis und A. profunda femoris, da meist eine ausreichende Kollateralisation über proximale Gefäßabschnitte vorhanden ist. Es gibt verschiedene Arbeiten, in denen die primäre Ligatur der Iliakalgefäße und der A. femoralis communis als erste Therapie der Wahl herangezogen wurde. Dabei wurde das amputationsfreie Überleben mit bis zu 99 % angegeben [15, 19]. Im untersuchten Patientenkollektiv wurde die primäre Ligatur bei 5 von 13 Patienten mit arterieller Gefäßbeteiligung durchgeführt (Tab. 2). Zwei der 5 Patienten entwickelten im stationären Verlauf eine Extremitätenischämie und wurden einer Revaskularisation mittels Veneninterponat unterzogen. In der Literatur wurde dieses Vorgehen bei progredienter Ischämiesymptomatik mit amputationsbedrohter Extremität ebenfalls berichtet [8, 14, 17]. Bei den übrigen primär ligierten Patienten lag der durchschnittliche Ankle-brachial-Index (ABI) bei 0,65 ± 0,24. Trotz klinisch kompensierter Hämodynamik sollte eine Ligatur aus unserer Sicht bei einem durchschnittlichen ABI von 0,65 nicht primär favorisiert werden.

**Table 4:** 

	Primär^c^ *n*
*Einbezogene Patienten*	12
*Ligatur der Vene*^a^	4
*Thrombektomie*	
Singulär	1
Zusätzlich Crossektomie	1
*Konservativ*^b^	6

^a^Ligatur der V. iliaca/femoralis communis/femoralis profunda oder femoralis superficialis

^b^Mittels therapeutischer Heparinisierung und testgerechter Antibiose

^**c**^Primäre Versorgung bei venöser Gefäßbeteiligung

Um einem ischämischen Verlauf und konsekutiven Extremitätenverlust vorzubeugen, favorisieren wir, wie auch andere Autoren, wenn möglich die primäre Revaskularisation des resezierten septischen Gefäßabschnitts [5, 6, 9, 22]. In unserem Patientenkollektiv erfolgte in 5 Fällen primär die arterielle Rekonstruktion unter Verwendung der ipsilateralen V. femoralis communis. In 2 Fällen wurde primär eine Patchplastik, einmal autologe Vene und einmal bovines Rinderperikard (XenoSure®, LeMaitre Vascular, Inc., Burlington, USA), auf das arrodierte Arteriensegment angelegt. In beiden Fällen kam es im Verlauf zu einer septischen Arrosionsblutung mit Patchausriss. Bei dem Patient mit Venenpatchplastik erfolgte in einer sekundären Operation die Rekonstruktion mittels V. femoralis communis, im anderen Fall bei fehlender geeigneter autologer Vene die arterielle Rekonstruktion mittels Allograft (MAQUET, INTERGARD SYNERGY, Durchmesser 8 mm). Grundsätzlich kann festgehalten werden, dass die Verwendung eines Patches auf Höhe des geschädigten Gefäßsegmentes in unserer Untersuchung nicht langfristig erfolgreich war (2 Blutungen bei 2 Anlagen) und das Risiko einer weiterführenden septischen Arrosion in dem betroffenen Segment hoch und nicht Erfolg versprechend ist.

Bei einem Patienten trat nach primärer Rekonstruktion durch Veneninterponat bei fortschreitendem Infektgeschehen eine septische Arrosionsblutung des autologen Graftmaterials auf. Hier musste eine sekundäre Ligatur auf Höhe der A. iliaca externa mit Resektion des Bypasses vorgenommen werden. Es zeigte sich hinterher keine kritische Extremitätenischämie.

Als Alternative zum autologen Gefäßersatz können extraanatomisch verlaufende Bypässe (z. B. Obturatorbypass), unter Umgehung des lokalen Infektes, angelegt werden [12, 20]. In unserem Kollektiv wurde primär bei einem Patienten ein alloplastischer Obturatorbypass angelegt. Dabei konnte ein Verschluss nach 220 Tagen festgestellt werden. Insgesamt konnte von den arteriellen Rekonstruktionen bei 5 von 8 Patienten ein Follow-up bestimmt werden. Die Offenheitsrate der gesamten arteriellen Rekonstruktionen betrug 87,5 % bei einem mittleren Follow-up von 421 Tagen. Dabei waren alle venösen Interponate im untersuchten Zeitraum perfundiert. Lediglich der Obturatorbypass zeigte sich verschlossen. In der Literatur werden Offenheitsraten von Obturatorbypässen mit 65 % bei einem Follow-up von 24 Monaten angegeben [4]. Grundsätzlich besteht bei nur einem angelegten Obturatorbypass keine suffiziente Aussagekraft, jedoch lässt sich in Zusammenschau der erhobenen Daten eine Tendenz zugunsten der autologen Gefäßrekonstruktion feststellen. Hier sind weitere Untersuchungen notwendig. Weiterhin sind alloplastische Gefäßrekonstruktionen aufgrund der niedrigen Compliance von Drogenpatienten durch erneute Injektionen bei fortgesetztem Drogenkonsum stark infektgefährdet [23]. Dieses Risiko ist bei den extraanatomischen Rekonstruktionen hoch, da diese bei subkutanen Verläufen leicht für weiterführende Drogeninjektionen missbraucht werden können [21]. Aus unserer Sicht sollte die Anlage eines alloplastischen extraanatomischen Bypass nur selektiv und übergangsweise zur sekundären Revaskularisation bei kritischer Extremitätenischämie erfolgen. In der Literatur wurde dieses Vorgehen zur Senkung der Amputationsrate bereits beschrieben [13]. Schlussendlich erscheint bei einer festgestellten Offenheitsrate von 100 % die Korrektur arterieller Blutungen unter Anlage eines autologen Interponats als aussichtsreich.

## Fazit für die Praxis

- Nach Differenzierung des Lokalbefundes (FKDS, CTA) ist unter kalkulierter Antibiose (β-Laktamase-stabil) eine breite Abszesseröffnung mit Exploration der Inguinalgefäße und offener Wundbehandlung durch einen in der Gefäßmedizin erfahrenen Chirurgen als Basismaßnahme erforderlich.
- Bei der Wundexploration ist im Sinne des Eigenschutzes auf den Ausschluss von Nadelfragmenten zu achten.
- Bei vaskulärer Beteiligung ist ein situationsgerechtes Vorgehen sinnvoll. Neben der Beseitigung komplizierter, septisch-venöser Thrombosen erscheint die Korrektur arterieller Blutungen mittels autologer Rekonstruktionsmaßnahmen aussichtsreich.
- Aufgrund der guten Kollateralisation der im Grunde gefäßgesunden Patienten ist als Ultima Ratio eine primäre Ligatur in Sondersituationen vertretbar.
